# Automatic Calibration of a Device for Blood Pressure Waveform Measurement

**DOI:** 10.3390/s23187985

**Published:** 2023-09-20

**Authors:** Rafał Siemasz, Krzysztof Tomczuk, Ziemowit Malecha, Piotr Andrzej Felisiak, Artur Weiser

**Affiliations:** 1 Faculty of Mechanical and Power Engineering, Wrocław University of Science and Technology, 50-370 Wrocław, Poland; rafal.siemasz@pwr.edu.pl (R.S.); piotr.felisiak@pwr.edu.pl (P.A.F.); 2 Department of Neurosurgery, Wrocław Medical University, 50-425 Wrocław, Poland; artur.weiser@umw.edu.pl

**Keywords:** blood pressure measurement, pneumatic sensor, blood pressure waveform, sensor calibration

## Abstract

This article presents a prototype of a new, non-invasive, cuffless, self-calibrating blood pressure measuring device equipped with a pneumatic pressure sensor. The developed sensor has a double function: it measures the waveform of blood pressure and calibrates the device. The device was used to conduct proof-of-concept measurements on 10 volunteers. The main novelty of the device is the pneumatic pressure sensor, which works on the principle of a pneumatic nozzle flapper amplifier with negative feedback. The developed device does not require a cuff and can be used on arteries where cuff placement would be impossible (e.g., on the carotid artery). The obtained results showed that the systolic and diastolic pressure measurement errors of the proposed device did not exceed ±6.6% and ±8.1%, respectively.

## 1. Introduction

Blood pressure (BP) waveform measurements enable a number of new indicators for the evaluation of hemodynamic changes in a patient. The application of the so-called transfer function additionally allows a non-invasive determination of the aortal wave and its indicators [1,2,3].

At present, there are many techniques and devices for noninvasive blood pressure measurement. Generally, the basic element of currently used devices is a measuring cuff, which allows one to determine approximate values of the systolic and diastolic pressure. Some of these instruments additionally enable the observation of the shape of the arterial pressure waveform, which can be regarded as one of the most important diagnostic parameters of the human cardiovascular system [4,5,6,7,8,9,10,11,12,13]. More advanced instruments also display real pressure values for observed waves [14,15,16,17,18,19,20,21,22]. The most well-known of these devices are Finapres (Finapres Medical Systems B.V., Enschede, The Netherlands), with its latest variants Portapres and Finometer, and Colin Pilot (DRE Medical, Inc., Louisville, KY, USA) [15,17,18,19].

Cuff-based blood pressure waveform monitors are increasingly being supplemented by devices based on noninvasive, electrical or pneumatic sensors. One potential advantage of noninvasive cuffless monitors is their ability to measure pressure waveforms in arteries, such as the subclavian artery, carotid artery, or superficial temporal artery, which are inaccessible to cuff-based devices. Sensors of this class of devices are usually available in electrical versions. Unfortunately, noninvasive sensors have their disadvantages, such as the need for calibration before each measurement. This is due to the fact that the ordinal values of the recorded BP waveform are represented as voltages, the dependence of which on BP can vary between measurements because it is affected by the stiffness of the artery and surrounding tissue. The most common calibration method is based on an inflatable pressure cuff surrounding the finger or arm. This can be a troublesome process for both patient and staff [21]. Although many indicators can be determined from blood pressure waveforms without information on the pressure scale (in cases where the pressure ratio is of interest), calibration remains a requirement if indicators are to be determined from the carotid artery wave. Solutions that are solely focused on the registration of the pulse wave of the blood without the actual pressure scale are described, for example, in [23,24,25].

The latest technical and methodological solutions for noninvasive medical diagnostic tools allow one to both measure real pressure values and show the shape of the arterial pressure wave without using a cuff [21,26,27,28,29,30,31,32,33,34,35].

One such interesting approach is based on the modified oscillometric principles, as described in [26]. The idea is that as the user presses a finger against the force and photoplethysmography (PPG) sensors installed in a mobile device, and the external pressure of the underlying artery is steadily increased while the device measures the applied pressure and the resulting variable-amplitude blood volume oscillations. The current visual feedback of the blood pressure wave as well as the calculated systolic and diastolic pressure are displayed on the device’s screen.

The concept of blood pressure estimation using mechanical plethysmography in connection with standard electrocardiography (ECG) is also noteworthy [31,32,33]. Signals from a novel, magnetoelastic skin curvature sensor (SC-sensor) and from the ECG are adaptively processed in order to estimate blood pressure according to a specifically established theoretical model based on two physiological parameters that serve as model input: the pulse wave transit time and an additional direct detection of mechanical oscillations of the carotid artery wall. As a result, the proposed device allows the continuous monitoring of blood pressure and is relatively convenient for the patient.

However, some of the cited articles [21,27,28,29] concerning the accuracy verification of cuffless BP waveform recording device readings clearly emphasize the need to calibrate the measuring device with a cuff each time before measuring a new subject.

This paper presents the next development stage of a new, noninvasive, cuffless blood pressure waveform measuring device equipped with a pneumatic pressure sensor. The prototype of this device was described in [11]. Since the measurement is carried out using pressurized air, a new concept was advanced, namely employing a pneumatic sensor to both measure the BP waveform and calibrate the device. For the purpose of device calibration, a modified oscillometric measurement principle and a model-based calibration characteristic were adopted.

## 2. Design and Operation of the Sensor

**Figure 1:**
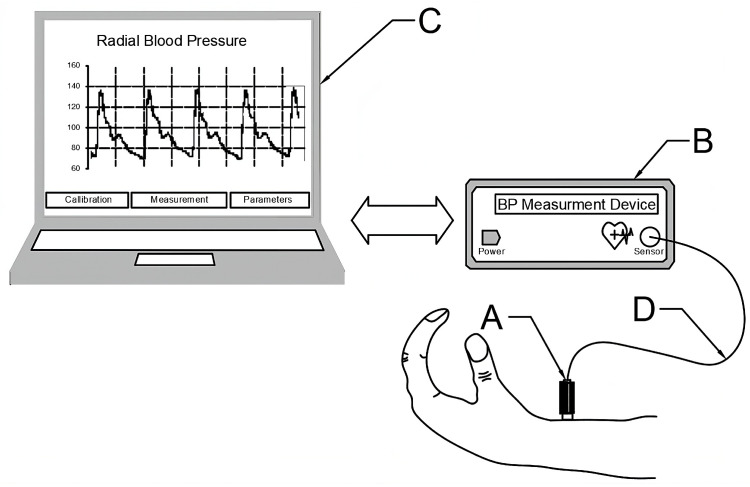
A device for blood pressure waveform measurement. A—pneumatic sensor, B—computer interface, C—notebook, D—elastic double-line pneumatic tube.

Figure 1 shows a sample BP waveform measurement in the radial artery obtained with a novel pneumatic sensor (A), computer interface (B), and computer (C). The sensor is connected to the interface via an elastic double-line pneumatic tube (D).

The operating principle of such a device is as follows: The sensor is placed against the patient’s body in a location where an artery with a palpable pulse runs directly below the skin, e.g., on the wrist, see Figure 1.

In the next step, under observation on a computer screen, the sensor is delicately pressed against the artery until the first slight pulsations are detected, and then it is secured in place as the fingers holding the sensor are placed on the patient’s body. At this stage, calibration starts, and the computer screen shows a pulse amplitude that initially increases and then decreases. The decrease indicates the end of calibration, and the BP waveform measurement can begin. The measurement is performed by pressing the sensor harder against the artery so that the BP wave amplitude appearing on the screen is likely the largest. This step concludes the measurement, and the screen displays both the waveform and its parameters.

Pneumatic BP waveform sensors are essentially nozzle-flapper amplifiers in which a rubber (silicone) membrane acts as a flapper and receives the measured pressure or force.

The function added to the sensor in its new version necessitates replacing the traditional nozzle-flapper amplifier [5] with so-called annulus venting. Amplifiers of this type are used in intraocular pressure sensors, for example, [36].

**Figure 2:**
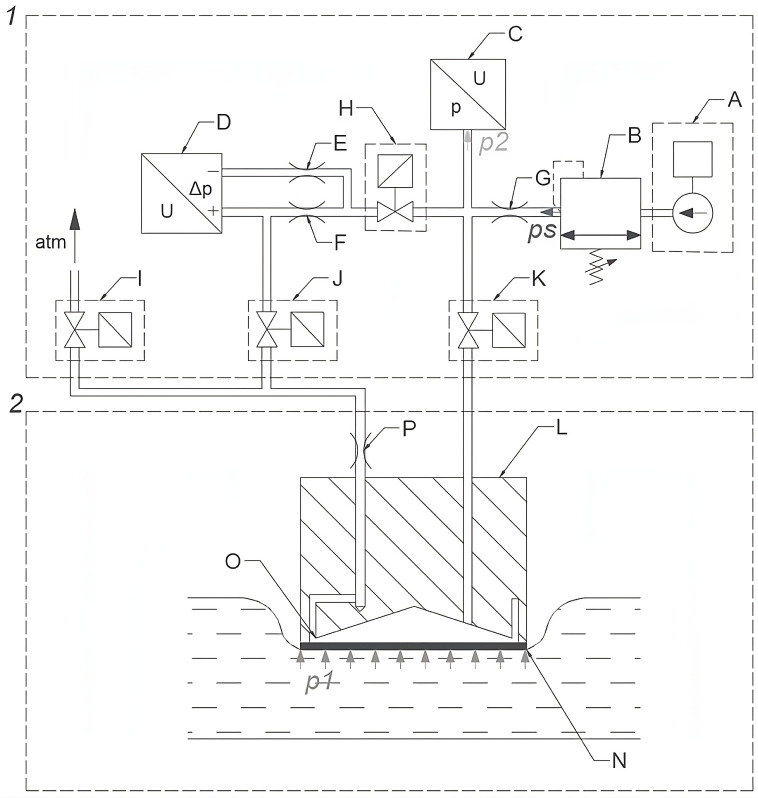
Sensor (2) with connected computer interface (1) and artery segment: A—compressor; B—pressure regulator; E, F, G—orifices; H, I, J, K—shut-off valves; L—sensor body; M—measurement chamber; N—membrane; O—venting gap; C—pressure transducer (Motorola MPX5050 [37]); D—differential pressure transducer (Nenutec 984m.333704 [38]); $p_{1}$—arterial pressure; $p_{2}$—pressure readout by the main pressure transmitter; $p_{s}$—output pressure from pressure regulator.

The complete measurement system can be seen in Figure 2. It consists of two main parts: the computer interface (1) and the sensor (2).

The interface (1) acts as an air supply, a processing system, and a control system. It includes a mini-compressor (A); a pressure stabilizer (B); a pressure transducer (C); a differential pressure transducer (D); orifices (E, F, and G); and shut-off valves (H, I, J, and K). Output pressures from transducers (C) and (D) are fed into a DAQ system.

**Figure 3:**
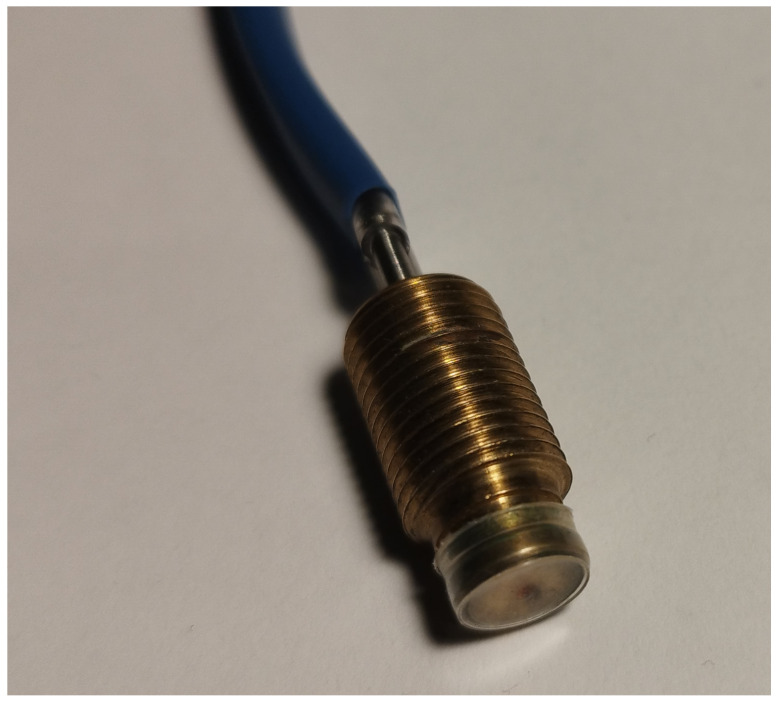
View of the pneumatic sensor without casing.

The sensor (2) includes a brass body (L), an orifice (P), a measurement chamber (M), a membrane (N), and a venting gap (O). The output pressure $p_{2}$ in the sensor is measured with a transducer (C), and the oscillations of the transducer’s membrane during calibration are measured with a differential pressure transducer (D). The sensor body can be seen in Figure 3.

The pneumatic capacity should be as small as possible to ensure the fastest possible response of the measurement system. The pneumatic tubes used are made of rigid silicone with an inner diameter of ø1 mm, and the wall thickness of the pneumatic tubes is 1 mm. The measuring diaphragm, with an active diameter of ø6.4 mm, is made of silicone certified for medical applications

During the BP waveform measurement, valves I and K have to be open, while valves J and H are closed. Supply air $p_{s}$ at a constant pressure flows from the pressure regulator (B) through the orifice (G) and reaches the measurement chamber (M). From there, it enters the venting gap (O) and exits to the atmosphere. The resistance of the venting gap (O) is variable because it depends on the position of the membrane (N) in relation to the edge of the measurement chamber (M).

As the measured pressure $p_{2}$ rises, the membrane (N) deflects, reducing the flow section of the venting gap (O). Consequently, the airflow through the sensor is reduced, and the pressure in the measurement chamber increases, preventing the further deflection of the membrane (N). The deflection stops when the value of pressure $p_{1}$ approximates the value of the measured pressure: $p_{2}\approx p_{1}$. This effect is due to negative feedback and also occurs when the measured pressure changes in time with a frequency value of up to 10 Hz. Owing to negative feedback and to the limited membrane deflection degree (in the order of 10 μm), the sensor’s static characteristic within the measurement range is linear.

During the sensor calibration, the valves H, J, and K, controlled via the control system, are in an open position, the sensor’s venting valve (I) is closed, the sensor with a closed venting valve is placed against the inspected artery, and the computer screen is observed. When regular pressure pulsations of the highest observable amplitude are visible on the screen, the compressor (A) is started and the stabilizer (B) increases the pressure $p_{s}$ in a linear manner, causing the sensor’s pulsation amplitude to initially rise. Pressure $p_{s}$ starts to gradually increase from zero to 40 kPa, resulting in the flattening of the artery. The oscillations (resulting from the movement of the membrane), which are visible on the computer screen, also initially rise and later decrease. These effects imply that the calibration process is finished.

The primary reason behind using an amplifier with annular venting is that the membrane in such a configuration can freely vibrate in response to artery movements without being restricted by a traditional nozzle-flapper amplifier.

This sequence of events indicates that the calibration is finished. The control system closes valves H and J and opens the sensor’s venting valve (I). The pressure stabilizer (B) increases and stabilizes the pressure $p_{s}$ at a value of 40 kPa. At this point, the BP wave can be measured.

## 3. Sensor Calibration Procedure

As noted above, the ordinate value of the plotted BP waveform is represented as the transducer’s output voltage, and the relationship between BP and this voltage, albeit linear, is not known [11]. This fact results from the need to flatten the examined artery using the sensor, while the force required to flatten the artery depends on the stiffness of both the artery and the surrounding tissue.

**Figure 4:**
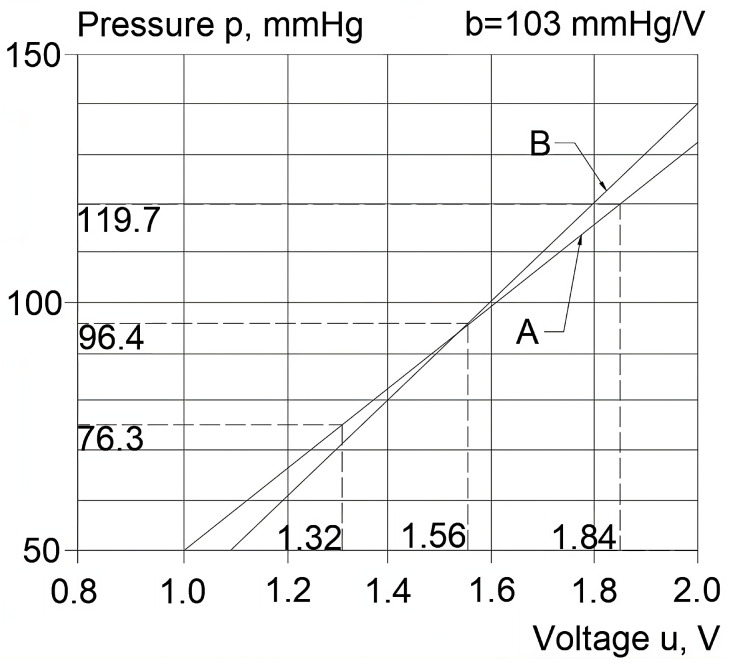
Individual characteristics determined using two methods: A—method based on the measurements of the systolic pressure $p_{s}$ and the diastolic pressure $p_{d}$ using a cuff monitor, B—method based on the oscillometric measurement of the mean BP values $p_{m}$ using the described device.

The relationship between BP and voltage, the so-called individual calibration characteristics, can be determined using two methods. The first, the traditional method, is based on prior measurements of systolic pressure $p_{s}$ and diastolic pressure $p_{d}$ and is normally performed using a cuff monitor [8]. These values, together with the voltage values—the maximum $u_{s}$ and minimum $u_{d}$, as read from the BP waveform curve—determine the individual calibration characteristic curve (see Figure 4), which can be described with the following equation of a line:

(1) p=a+bu

where *p* is blood pressure in mmHg, *u* is voltage in V, *a* is the initial calibration value in mmHg, $a=p_{s}-bu_{s}$, and *b* is the calibration slope in mmHg/V,

(2) $$b=\frac{p_{s}-p_{d}}{u_{s}-u_{d}}.$$

Individual characteristics can be plotted based only on specific variable values, e.g., $p_{s}$ = 119.7 mmHg, $p_{d}$ = 76.3 mmHg, $u_{s}$ = 1.84 V, and $u_{d}$ = 1.32 V (see Figure 4).

The second method, which is actually used in the device described herein, consists of the oscillometric measurement of the mean BP values in the examined artery $p_{m}$ and requires the calibration slope *b* to be known (Figure 4). The oscillometric measurement of mean BP values is a method commonly used in cuff monitors in which the oscillations of an increasing or decreasing test pressure are significant enough to be measured with a single transducer [8]. In the case of this sensor, however, the amplitude of the air pressure oscillations in the measurement chamber (M) (Figure 2) is limited, and its measurement requires a custom-built differential pressure transducer (D) [11].

**Figure 5:**
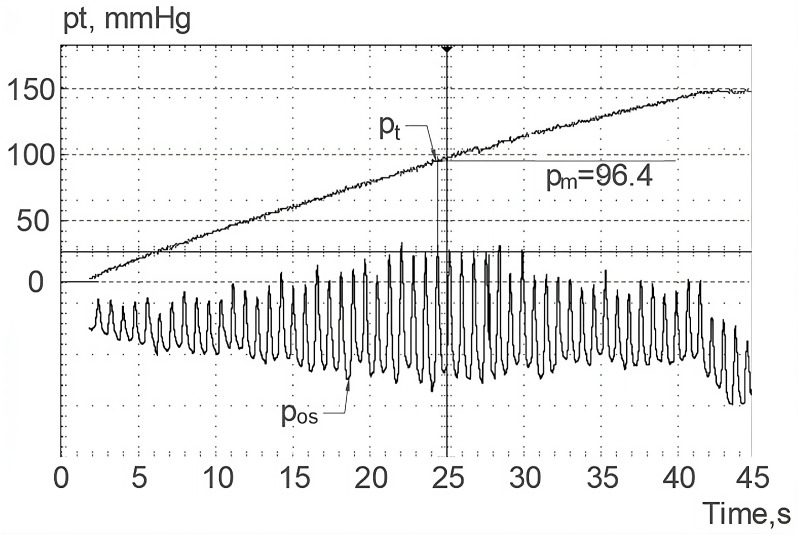
Oscilloscope printout of the pressure in the sensor measurement chamber: $p_{t}$—test pressure, $p_{os}$—oscillatory component of the test pressure.

Figure 5 shows a plot of test pressure $p_{t}$ and pressure oscillation $p_{os}$ in a volunteer subject. The maximum amplitude of these oscillations was observed at the moment when the test pressure, which flattened the artery, approximated the mean BP value.

**Figure 6:**
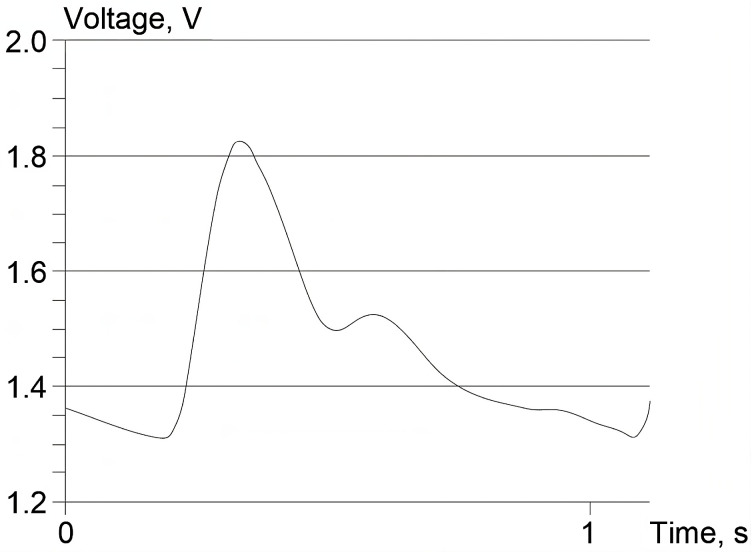
BP waveform curve of a volunteer.

The test pressure value ($p_{m}$ = 96.4 mmHg here), together with the calculated mean voltage value for the recorded BP waveform in an examined volunteer $u_{m}$ = 1.56 V (see Figure 6), defines one point of the individual calibration characteristics (Figure 4). The definition of actual calibration plot behavior, however, also requires the calibration slope *b*.

This slope is defined by the transduction coefficients of the sensor $k_{1}$ and the measurement transducer $k_{2}$, while

(3) $$k_{1}=\frac{\Delta p_{2}}{\Delta p_{1}}=\frac{A_{1}}{A_{2}},$$

(4) $$k_{2}=\frac{\Delta u}{\Delta p_{2}}$$

where $A_{1}$ and $A_{2}$ are membrane areas on the side of pressures $p_{1}$ and $p_{2}$, respectively (Figure 2); $\Delta p_{1}$ and $\Delta p_{2}$ are pressure increments of $p_{1}$ and $p_{2}$, respectively; and Δu is the transducer voltage increment.

If the measured pressure $p_{1}$ acts over the whole surface $A_{1}$, then $A_{1}\approx A_{2}$ and $k_{1}\approx 1$. However, in BP waveform measurements, only part of the $A_{1}$ area is subjected to the pressure in the pulsating artery. If it is assumed that the average width of a flattened radial artery is s=4 mm and the membrane diameter is d=6.5 mm, then $A_{2}=\frac{\pi d^{2}}{4}\approx 33$ mm^2^ and $A_{1}\approx sd\approx 26$ mm^2^, while $k_{1}\approx 0.79$. At the same time, $k_{2}=12\frac{\mathrm{mV}}{\mathrm{mmHg}}$.

Slope *b* in (1) corresponds to the $\frac{\Delta p_{1}}{\Delta u}$ relation. Considering (3) and (4), its value can be calculated from

(5) $$b_{c}=\frac{1}{k_{1}k_{2}}=105.5\frac{\mathrm{mmHg}}{V}.$$

The calculated value of slope $b_{c}$ was verified in statistical examinations performed on volunteers of both sexes with ages between 25 and 76. First, according to the international protocol for the validation of blood pressure measuring devices in adults (the auscultation of Korotkoff sounds by two operators simultaneously) [39], the systolic pressure $p_{s}$ and diastolic pressure $p_{d}$ of each subject were measured. In the next step, the device was used to record the BP waveform, which provided the wave’s maximal voltage $u_{s}$ and minimal voltage $u_{d}$. This method was used to prepare 45 individual calibration characteristics with various calibration slopes. The slopes’ mean value was $b_{m}=100.4\frac{\mathrm{mmHg}}{V}$. As the two calculated values of slope $b_{c}$ and $b_{m}$ varied by no more than 5%, the assumed mean value was $b=103\frac{\mathrm{mmHg}}{V}$. The final individual calibration characteristic, calculated using the proposed method, can be described by

(6) $$p=p_{m}+b\left(u-u_{m}\right)=p_{m}+103\left(u-u_{m}\right).$$

## 4. Preliminary Tests on Volunteers

A preliminary test of the device was performed with the help of ten volunteers; the test was by no means intended as a scientifically rigorous experiment, but it would provide a proof of concept and simple verification, whether the solution was promising or not. The proposed calibration method for the device discussed in Section 3 was compared with the Erkameter 125 Pro wrist cuff monitor (ERKA, Bad Tolz, Germany). The comparison consisted in assigning individual characteristics to each of the subjects. These characteristics were used to determine the pressure in two of the characteristic points on the subject’s BP waveform. These points were chosen to be the systolic and diastolic pressure. First, the subject’s systolic and diastolic pressures were measured three times using a wrist cuff monitor. The mean $p_{s}$ and $p_{d}$ values are listed in Table 1.

Next, the device was used to measure the mean BP value $p_{m}$ and record the waveform behavior, which in turn served to determine the voltages $u_{s}$, $u_{d}$, and $u_{m}$. These data made it possible to describe the calibration characteristics of each subject using (6), which yielded the calculated systolic pressure $p_{sc}$ and the calculated diastolic pressure $p_{dc}$, i.e.,

(7) $$p_{sc}=p_{m}+b\left(u_{s}-u_{m}\right),$$

(8) $$p_{dc}=p_{m}+b\left(u_{d}-u_{m}\right),$$

as well as their relative difference

(9) $$\Delta p_{s}=\frac{p_{s}-p_{sc}}{p_{s}}\cdot 100\%,$$

(10) $$\Delta p_{d}=\frac{p_{d}-p_{dc}}{p_{d}}\cdot 100\%.$$

At this stage of the research, ten volunteers were subjected to an examination for several days, and the 10 obtained measurement results are listed in Table 1. As can be seen, the relative differences between measured and calculated pressure values did not exceed ±9%. The issue of the reliability and repeatability of these methods remains a subject for further investigation in the next stage of the research (i.e., in clinical tests on patients).

## 5. Conclusions and Future Work

Current medical diagnostic instruments that allow the measurement of blood pulse wave parameters and the simultaneous presentation of the pulse wave shape require the use of a sensor that records the pulse wave shape (such as a photoplethysmograph) along with a measurement cuff to determine the pressure scale. Hence, the concept emerged of a pneumatic BP waveform sensor serving a double function: measuring the BP waveform and calibrating the device. Using the unique features of a pneumatic sensor, it was possible to develop a prototype of an instrument that has most of the advantages of the best devices in its class and does not require measuring two different physical quantities (e.g., force and light absorption coefficient).

Preliminary tests on volunteers proved the concept to be correct and highlighted an additional advantage of the device. The results obtained from the preliminary comparative measurements on 10 volunteers indicated that the errors in the measurement of systolic and diastolic blood pressure with the proposed device did not exceed, respectively, 6.6% and 8.1%.

The new design does not require one to center the sensor very carefully over an artery; this is a significant advantage over sensors with the traditional nozzle-flapper design.

Since the cuff-based measurements introduced significant errors, in our opinion, comparisons should be performed relative to the most accurate method, i.e., the direct, invasive measurement of blood pressure. Then, referring to the invasive measurements, the accuracy of the cuff-based method and our method can be compared quite reliably. We foresee this as future work. Further research will focus on improving the described method of instrument calibration. It will include the development of a special band for the steady positioning of the sensor and long-term blood pressure monitoring.

The developed device is potentially intended for use mainly as equipment for general practitioners, cardiologists, and people subject to heart or circulatory system diseases.

## Figures and Tables

**Table 1:** Preliminary evaluation of the new calibration method.

Subject	$p_{s}$	$p_{m}$	$u_{s}$	$u_{m}$	$p_{\mathrm{sc}}$	$\Delta p_{s}$
	$p_{d}$		$u_{d}$		$p_{dc}$	$\Delta p_{d}$
	mmHg	mmHg	V	V	mmHg	%
1	131.9	91.2	1.70	1.32	130.3	1.2
	76.7		1.14		72.7	5.3
2	122.8	95.9	1.92	1.58	130.9	−6.6
	76.3		1.33		70.2	8.1
3	127.7	99.5	1.86	1.55	131.4	−2.9
	83.0		1.38		82.0	1.2
4	126.6	91.5	1.69	1.38	123.4	2.5
	80.2		1.25		78.0	2.6
5	143.0	107.0	1.90	1.52	146.1	−2.2
	92.0		1.34		88.5	3.9
6	128.8	100.1	1.65	1.43	122.8	4.7
	83.1		1.20		76.4	8.1
7	131.4	106.0	1.67	1.37	136.9	−4.2
	65.0		1.02		70.0	−7.6
8	119.7	96.4	1.84	1.56	125.2	−4.6
	76.3		1.32		71.7	6.1
9	118.9	93.7	1.45	1.16	123.6	−3.9
	79.2		1.05		82.4	−4.0
10	122.5	96.2	1.55	1.22	130.2	−6.3
	80.4		1.13		86.9	−8.1

## Data Availability

The data presented in this study are available on request from the corresponding author. The data are not publicly available due to ethical reasons.

## Abbreviations

The following abbreviations are used in this manuscript:

BP	Blood pressure
